# Draft Genome Sequence of the Ale-Fermenting *Saccharomyces cerevisiae* Strain GSY2239

**DOI:** 10.1128/genomeA.00776-15

**Published:** 2015-07-23

**Authors:** Jana M. U’Ren, Jennifer H. Wisecaver, Andrew L. Paek, Barbara L. Dunn, Bonnie L. Hurwitz

**Affiliations:** a School of Plant Sciences, University of Arizona, Tucson, Arizona, USA; b Department of Ecology and Evolutionary Biology, University of Arizona, Tucson, Arizona, USA; c Department of Molecular and Cellular Biology, University of Arizona, Tucson, Arizona, USA; d Department of Genetics, Stanford University, Stanford, California, USA

## Abstract

*Saccharomyces cerevisiae* strain GSY2239 is derived from an industrial yeast strain used to ferment ale-style beer. We present here the 11.5-Mb draft genome sequence for this organism.

## GENOME ANNOUNCEMENT

*Saccharomyces* spp. have been utilized by humans to brew beer for >7,000 years (1). Over this time period, domesticated strains have evolved that differ in their fermentation characteristics, alcohol tolerance, fermentation temperature, sugar utilization, and flavor compounds (2, 3). In general, there have been fewer molecular or genomic studies of brewing yeasts than those of other yeast strains, because brewing yeasts are typically polyploid or allopolyploid, contain high levels of heterozygosity, and have low spore viabilities (3–5). However, recent comparative genomic studies of wine and ale yeasts have demonstrated that selection for specific physiological properties during fermentation can result in gene duplication, chromosomal rearrangements, interspecific hybridization, horizontal gene transfer, and high levels of sequence variation (6–10).

GSY2239 was derived from the industrial brewing strain Wyeast1388 (Wyeast Laboratories). Diploid spores of Wyeast1388, an *HO+* (homothallic) tetraploid *Saccharomyces cerevisiae* strain, were isolated using tetrad microdissection (11). A single spore was chosen for an additional round of meiosis and self-mating to minimize heterozygosity (to make genome assembly easier), yielding isolate GSY2239. Fluorescence-activated cell sorting (FACS) using known reference strains confirmed GSY2239 to be a tetraploid.

To minimize mitochondrial DNA sequences, GSY2239 was plated on yeast extract-peptone (YEP)-glucose containing 30 µg/ml ethidium bromide (12) to induce petite mutations. After 2 days, multiple colonies were isolated, and lack of growth on a nonfermentable substrate (YEP-glycerol) was confirmed. For genomic DNA extraction, a single colony was grown overnight in 3 ml of YEP plus 2% dextrose. Cells were then resuspended in 200 µl of Triton X-100, 1% SDS, 100 mM NaCl, 50 mM Tris-Cl (pH 8.0), and 1 mM EDTA (pH 8.0), whereby DNA was extracted using phenol:chloroform and resuspended in 200-µl of Tris EDTA (TE) buffer with RNase A (50 µg/ml).

Shotgun genome sequencing was performed using the GS-FLX Titanium platform (454 Life Sciences, Roche, Mannheim, Germany) at the University of Arizona Genetics Core. Sequences were filtered to eliminate the resulting low-quality data (13), and the 491,192 high-quality reads remaining were assembled into contigs *de novo* using the Newbler assembler (454 Life Sciences), obtaining approximately 15-fold genome coverage. The GSY2239 genome displayed very low levels of heterozygosity (<0.004%); thus, sequences were assembled as for a single haplotype genome. An 11.5-Mb high-quality assembly with 578 contigs was obtained, corresponding to 98% of the *S. cerevisiae* S288C nuclear genome, with an average contig length of 20,313 bp, a maximum contig length of 219,755 bp, and an *N*_50_ contig length of 60,719 bp, for a total of 11,537,987 residues. Using the *S. cerevisiae* training set, the gene finder Augustus (14) identified *ab initio* 5,365 complete protein-coding genes on repeat-masked scaffolds (RepeatMasker [15]). A total of 5,354 of these genes had top BLAST hits to either *S. cerevisiae* (*n* = 5,341) or other *Saccharomyces* spp., including *Saccharomyces carlsbergensis* and *Saccharomyces pastorianus* (*n* = 13) (BLASTp *E* value, 1 × 10^−3^). The remaining 11 genes had top BLAST hits to other fungi and metazoans.

### Nucleotide sequence accession numbers.

This whole-genome shotgun project has been deposited in DDBJ/ENA/GenBank under the accession no. LDOE00000000. The version described in this paper is the first version, LDOE00000000.1.
